# Survival and Control Prognosticators of Recurrent Gynecological Malignancies of the Pelvis and Para-aortic Region Treated with Stereotactic Body Radiation Therapy

**DOI:** 10.3389/fonc.2016.00249

**Published:** 2016-11-22

**Authors:** Shaakir Hasan, Anthony Ricco, Kaylette Jenkins, Rachelle Lanciano, Alexandra Hanlon, John Lamond, Jun Yang, Jing Feng, Michael Good, Joel Noumoff, Luther Brady

**Affiliations:** ^1^Radiation Oncology, Philadelphia Cyberknife, Crozer-Keystone Healthcare System, Havertown, PA, USA; ^2^University of Pennsylvania School of Nursing, Philadelphia, PA, USA

**Keywords:** gynecological malignancies, stereotactic body radiation therapy, endometrial neoplasms, uterine cervical neoplasms, ovarian neoplasms, uterine neoplasms

## Abstract

**Purpose:**

To define prognostic factors associated with improved survival and local control (LC) for gynecologic cancer recurrences limited to the pelvis and para-aortic (PA) region using stereotactic body radiation therapy (SBRT).

**Methods:**

Between 2/2008 and 7/2014, 30 women (35 targets) with pelvic or PA recurrence of endometrioid (*n* = 12), cervical (*n* = 11), ovarian (*n* = 3), uterine-serous (*n* = 2), or carcinosarcoma (*n* = 2) cancer were treated with SBRT. Eleven recurrences were located in the central pelvis, 11 along the pelvic sidewall (PSW), and 13 in the PA region.

**Results:**

Five-year survival for all patients was 42% with a median survival of 43.4 months. Multivariate analysis revealed better performance status (PS), and smaller clinical tumor volume was significant for improved survival (*p* < 0.05).

**Conclusion:**

SBRT is a local therapy for recurrent gynecological malignancies in the pelvis and PA region with curative potential. SBRT is especially effective for LC when targeting PSW or PA recurrence and for patients with a cervical/endometrioid uterine primary. Survival is improved for patients with better PS and smaller recurrence volume prior to SBRT.

## Introduction

An estimated 30–40% of gynecological malignancies recur after initial treatment, leaving many patients with a poor prognosis and limited treatment options (1). Regional failures of endometrial, cervical, and ovarian cancer frequently include areas of prior radiation therapy (RT) used for initial treatment such as pelvic sidewall (PSW) and para-aortic (PA) lymph node regions (2). As such, potential morbidity does not permit re-irradiation at effective doses with conventional techniques. Alternatively, central pelvic recurrences can be treated surgically; however, many patients are inoperable in particular those with nodal recurrences. Chemotherapy has proven to be a less effective salvage treatment with limited palliation and no chance for long-term survival. Stereotactic body radiation therapy (SBRT) is emerging as an attractive primary option for recurrent tumors in the pelvis and PA region, because the highly conformal and precise radiation delivery offers a local non-surgical salvage option, even in the setting of re-irradiation (3).

Several retrospective series have reported pelvic exenteration and lymph node dissection as an option for highly selected patients with local gynecologic recurrence (4). However, survival data widely varies and the procedures are morbid, with grades 3–5 post-operative complications as high as 44% (5). For inoperable patients, local and pelvic lymph node recurrences have been treated with conventional external beam radiotherapy (EBRT) to the pelvis, with or without abdominal radiation and systemic therapy. In the setting of prior pelvic irradiation, intensity modulated radiotherapy is often employed in order to limit dose to previous treatment fields, though the total dose is still limited. Ablative doses in the re-irradiation setting can be reached with brachytherapy; however, recurrence must be at the vaginal cuff. Like surgery, the outcomes from retrospective series of EBRT and brachytherapy are inconsistent, likely due to selection bias (6–9). Chemotherapy alone is typically reserved as palliation for metastatic disease or in combination with surgery or radiation with curative intent for locoregional recurrences. In either scenario, platinum-based systemic therapy is typically used alone or in combination with other agents (10–14).

Potentially curative therapy with radiation for pelvic and PA recurrence after initial therapy has been limited to patients not previously irradiated due to the potential toxicity to organs at risk (OAR) including bowel and bladder by exceeding tolerance doses to these organs. SBRT potentially minimizes this toxicity by precise tracking of the tumor with tight margins which allows high doses of radiation to be delivered to the target while relatively sparing nearby OAR that received prior RT. For those patients who have not received previous radiation, SBRT may be even more effective since it can be used as a boost with EBRT with implant-like dose distribution for difficult to implant areas such as PSW and PA recurrences. Among the technology equipped to deliver stereotactic radiotherapy, CyberKnife (Accuray Inc., Sunnyvale, CA, USA) is a robotic radiosurgery system in which a compact 6 MV photon linear accelerator mounted on a robotic arm delivers hundreds of radiation beamlets to the target from different angles as designed by the planning computers. A high intensity dose is delivered to the tumor and the dose to the surrounding tissue falls off faster than with conventional radiation. The system takes a pair of orthogonal X-ray images every three to five beams during SBRT to track the target’s position and the robot will compensate for the target’s motion to achieve overall precision of 1 mm or better. The sub-mm precision allows tighter margins than conventional radiation (15), and is well suited for re-irradiation, as previous prospective trials demonstrated no increased toxicity when SBRT was delivered to sites previously treated with conventional RT (16–18).

Evidence suggests that an SBRT boost is a viable alternative to brachytherapy boost for patients with primary cervical or endometrial tumors who were not amenable to brachytherapy (19, 20). Recurrences treated with SBRT are described in the literature, albeit with a small or heterogenous patient population. Some studies combine the results of primary and recurrent tumors (21, 22), others include non-gynecologic primaries (23–26), and some have less than 10 patients (27–29). Guckenberger et al. reported an 81% 3-year local control (LC) rate and median overall survival of 25 months in (mostly central) pelvic recurrences treated with EBRT and an SBRT boost (30). We report our single institutional experience of SBRT for recurrent gynecologic malignancies in the pelvic and PA region to explore the rate of long-term LC that may ultimately translate into a potential cure.

## Materials and Methods

### Patient Characteristics

Between February 2008 and July 2014, 30 patients (35 lesions) were treated with SBRT for gynecologic malignancies with locoregional recurrence in either the central pelvis (CP), along the PSW, or para-aortic nodes (PAN) in this IRB approved study. Recurrences were diagnosed by pelvic exam, computed tomography (CT), magnetic resonance imaging (MRI), and/or positron emission tomography (PET)–CT. Every patient had radiographic follow-up at least 3 months after treatment with SBRT. Palliative and metastatic patients were excluded from the study. The median age was 70 years old (37–89) and median time to recurrence from initial diagnosis was 28 months (3–507 months). Targets included 13 PAN, 11 CP, and 11 PSW recurrences. Of the 30 women, 12 had endometrioid adenocarcinoma of the uterus, 11 squamous cell carcinoma of the cervix, 3 papillary serous adenocarcinoma of the ovary, 2 papillary serous adenocarcinoma of the uterus, and 2 carcinosarcoma (mixed Mullerian tumor) of the uterus. The median tumor size was 3.3 cm, ranging from 0.9 to 9.1 cm. Fifteen of the 35 recurrences were in areas previously irradiated as part of the initial treatment. Twenty-four of 30 patients received surgery for their initial treatment and 9 received chemotherapy. Platinum-based chemotherapy was used as salvage therapy in conjunction with SBRT in 13 of 29 patients (unknown *n* = 1). The median follow-up duration was 24 months (5–92 months). A summary of patient characteristics and treatment is summarized in Table 1.

**Table 1:** **Patient and treatment characteristics**.

Pt No	Tumor No	Age	Initial Dx	Location of rec	Time to rec (mo)	CTV (cc)	Chemo for rec	Previous XRT	SBRT delivery	Total BED Gy10^d^	Local control	Survival	Grade 3/4 Tox	F/U (mo)
1	1	58	Ovarian-serous	PSW	28	52.9	No	No	CK only	59.5	LR	Death	No	17
2	2	70	Endometrial	Central	19	29.1	Yes	No	CK boost	81.1	LR	Death	No	15
3	3	83	Endometrial	PA	15	32.8	No	Yes	CK only	35.7	LC	Death	No	6
4	4	82	Endometrial	PA	38	12.7	Yes	No	CK only	72	LC	Alive	No	92
5	5	80	Cervical	PSW	3	0.91	No	No	CK boost	112.8	LC	Death	No	19
6	6	40	Cervical	Central	28	9.4	Yes	No	CK boost	83.7	LC	Alive	No	24
7	7	72	Cervical	Central	78	29.8	Yes	No	CK boost	90.6	LC	Alive	No^a^	45
8	8	82	Cervical	Central	507	285.3	No	Yes	CK only	48	LC	Death	No	5
9	9	68	Ovarian-clear cell	PSW	54	30	No	No	CK only	42.6	LC	Death	No	29
10	10	79	Cervical	PSW	15	68.3	No	Yes	CK only	48	LC	Death	No	7
11	11	37	Cervical	Central	29	88.8	Yes	Yes	CK only	37.5	LC	Alive	**Yes**^b^	21
12	12	64	Endometrial	PSW	18	8.3	No	No	CK boost	75.6	LC	Alive	No^c^	77
13	13	72	Endometrial	PA	6	1.8	No	No	CK boost	81.1	LC	Alive	No	57
14	14	62	Endometrial	PA	3	30.7	No	No	CK only	37.5	LC	Death	No	63
–	15	62	Endometrial	PA	3	8.4	No	No	CK only	37.5	LC	Death	No	–
15	16	71	Cervical	PA	10	8.3	Yes	No	CK only	65.6	LC	Alive	No	63
16	17	70	Endometrial	PA	5	103.5	Yes	No	CK boost	72.6	LC	Alive	No	60
–	18	73	Endometrial	PA	45	7.7	No	Yes	CK only	65.6	LC	Alive	No	–
17	19	76	Carcinosarcoma	Central	14	14.9	No	Yes	CK only	59.5	LR	Alive	No	23
18	20	80	Endometrial	Central	10	85.7	Yes	Yes	CK only	37.5	LC	Death	No	11
19	21	80	Cervical	Central	34	8.3	–	No	CK boost	98	LC	Alive	No	8
20	22	89	Uterine-serous	PA	34	24	No	No	CK only	72	LR	Death	No	16
–	23	89	Uterine-serous	PA	38	8.7	No	No	CK only	48	LR	Death	No	–
21	24	65	Carcinosarcoma	PA	35	31.2	Yes	No	CK only	37.5	LC	Alive	No	22
22	25	60	Uterine-serous	Central	47	50	No	Yes	CK only	48	LR	Death	No	14
–	26	60	Uterine-serous	Central	47	11.4	No	Yes	CK only	48	LR	Death	No	–
23	27	89	Endometrial	PSW	229	38.4	No	Yes	CK only	72	LC	Death	No	43
24	28	79	Endometrial	Central	25	15.7	No	Yes	CK only	59.5	LR	Alive	No	29
25	29	66	Ovarian-serous	PSW	80	4.7	Yes	No	CK only	48	LR	Alive	No	47
26	30	59	Endometrial	PSW	153	43.6	Yes	No	CK boost	81.1	LC	Alive	No	64
27	31	52	Cervical	PSW	3	36.7	No	Yes	CK only	43.2	LC	Death	No	59
–	32	52	Cervical	PSW	3	36.7	No	Yes	CK only	43.2	LC	Death	No	–
28	33	56	Cervical	PA	30	67.3	Yes	Yes	CK only	53.6	LC	Alive	No	17
29	34	60	Endometrial	PSW	98	86.5	Yes	Yes	CK only	48	LC	Alive	No	10
30	35	66	Cervical	PA	21	7.9	No	No	CK only	48	LC	Death	No	26

*Pt, patient; No, number; Dx, diagnosis; rec, recurrence; mo, months; CTV, clinical target volume; cc, cubic centimeters; Chemo, chemotherapy; BED, biological equivalent dose; XRT, radiotherapy; Tox, toxicity; PA, para-aortic; PSW, pelvic sidewall; CK, cyberknife; LC, local control; LR, local recurrence; F/U, follow-up*.

*^a^Grade 2 radiation cystitis*.

*^b^Grade 3 rectovaginal/vaginovesicular fistulas*.

*^c^Grade 2 proctitis*.

*^d^Includes all radiation delivered for recurrence which includes SBRT alone dose or external beam + SBRT combined doses if boost used*.

*–, unknown if chemotherapy delivered*.

### SBRT Treatment

Nine of the 35 lesions were treated with EBRT followed by an SBRT boost to gross tumor volume (GTV) and the remaining 26 targets were treated with SBRT alone, 15 of which could not receive EBRT as salvage therapy because of previous irradiation. GTV = clinical tumor volume (CTV) ranged from 0.9 to 285.3 cc (median 29.8 cc) and the median SBRT dose for all patients was 27.5 Gy (15–40 Gy) in three to five fractions (median five fractions). Patients who received EBRT with an SBRT boost for recurrent tumors received a biological equivalent dose (BED) of 72.6–112.8 Gy (median 81.1 Gy) to the target lesions, whereas the BED for patients who only received SBRT received 35.7–72 Gy (median 48 Gy). Dose was prescribed to the 60–74% (median 69%) isodose line.

Tumors were tracked with fiducials (*n* = 23) or X-site spine tracking algorithm (*n* = 12). The GTV was defined as visible tumor on CT with oral and IV contrast, MRI, and PET–CT with images merged for target definition. The planning target volume (PTV) included the GTV and typically a 3-mm margin (range 0–5 mm) except at critical contiguous structures such as bowel or the aorta. A pair of orthogonal X-rays were acquired during the setup, and aligned with DRR (digital radiographs reconstructed from the planning CTs) using spine/hip or fiducial as match landmark. Based on the alignment, the treatment table is remotely moved to shift patient to the desired treatment position and new X-ray taken to verify an appropriate match prior the treatment. Once treatment starts, X-rays are taken every three to five beams throughout treatment delivery and robot will offset linac position based on the X-rays alignment to track patient’s position and achieve optimal targeting precision. Our Cyberknife treatment uses 57–201 (median 145) non-isocentric 6-MV photon beams to irradiate a single target stereotactically.

### Assessment of Results

All patients had at least 3 months of radiographic follow-up with pelvic exam and most recent imaging within at least 6 months of the latest clinical follow-up. Local recurrence after salvage therapy was defined as a lesion that developed within the SBRT PTV. Any tumor that appeared outside the radiation target, including non-irradiated PSW or PANs were considered distant failures. Radiation-induced toxicity was based on the Common Terminology Criteria for Adverse Events (CTCAE) version 3.0, and attributed to RT if the toxic event occurred in the absence of progressive disease (31). Overall survival and LC estimates for the total cohort were generated using log-rank Kaplan–Meier methodology, with survival and LC comparisons based on the Wilcoxon method. Unadjusted and adjusted multivariate hazard ratios for prognostic factors were estimated using Cox Proportional Hazard modeling. All statistics were generated *via* MedCalc software, version 16.8.

## Results

### Survival

**Figure 1:**
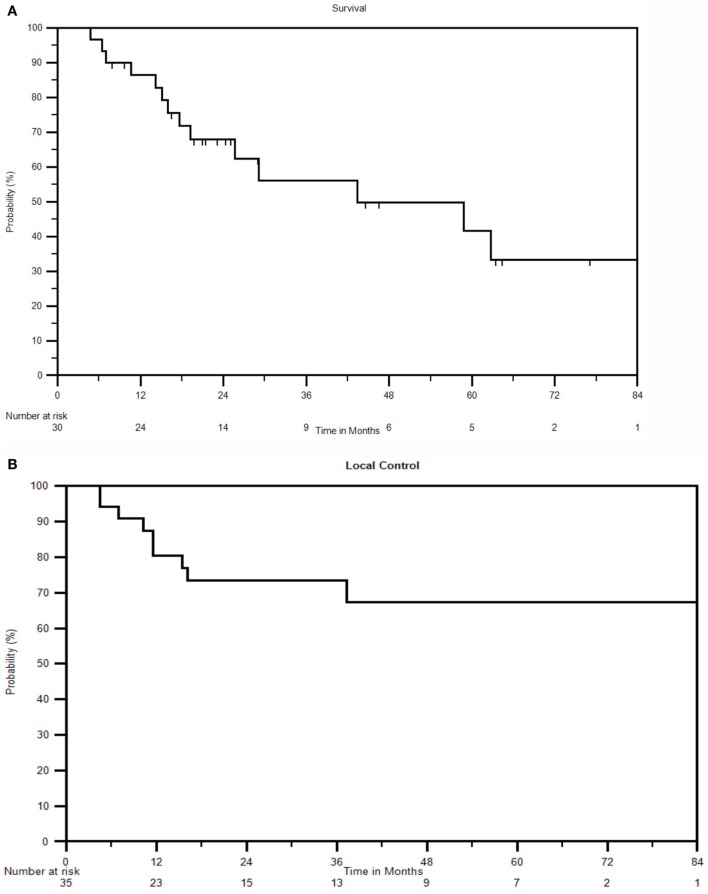

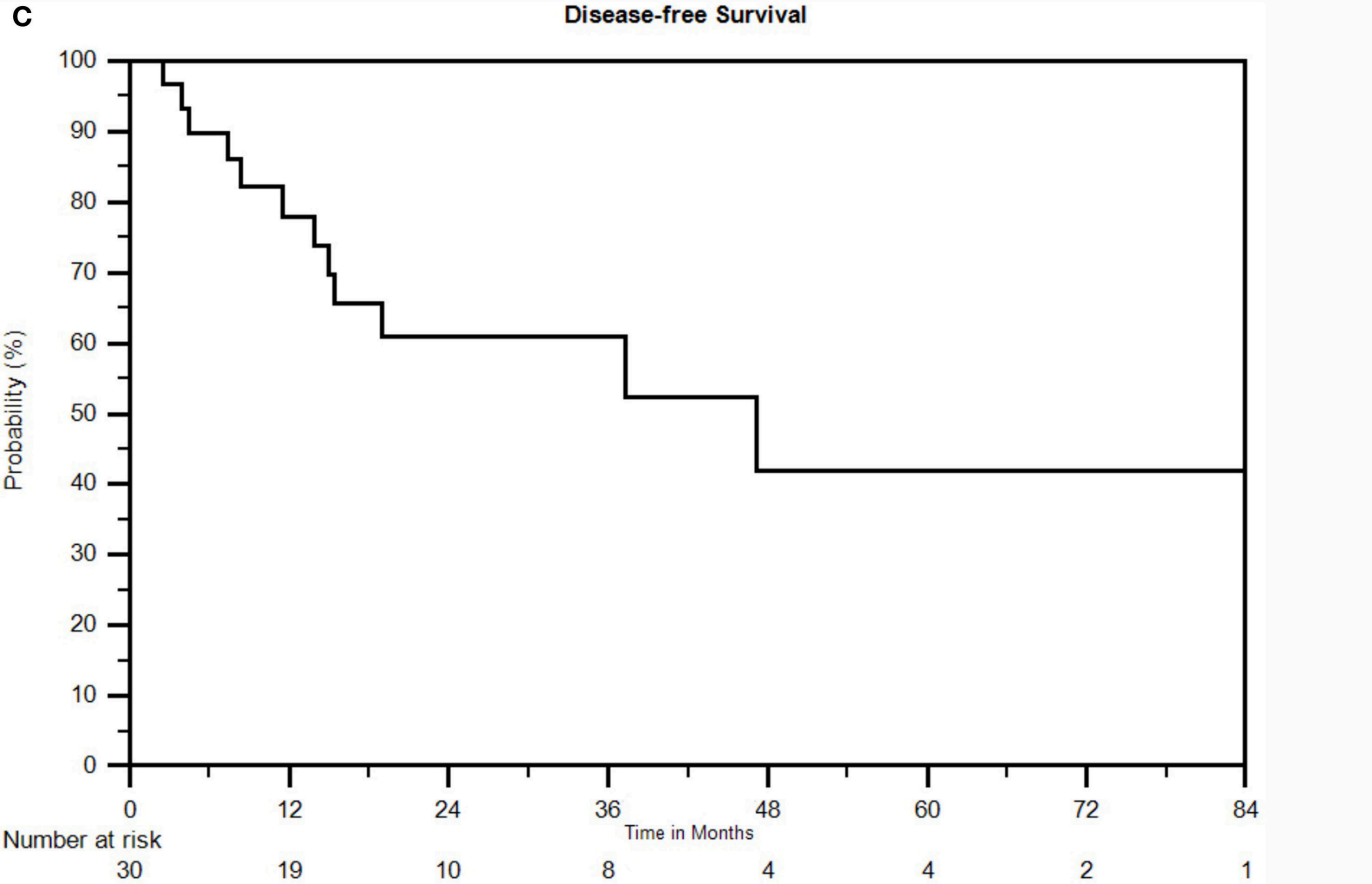
**(A)** Kaplan–Meier survival curve for all patients. **(B)** Kaplan–Meier local control curve for all recurrences treated with SBRT. **(C)** Kaplan–Meier disease-free survival curve for all patients.

The 5-year OS for all patients was 42%, with a median overall survival of 43.3 months. At last follow-up, 16 of 30 (53%) patients were alive. At 1, 2, 3, and 4 years, the OS was 85, 69, 56, and 49%, respectively (Figure 1A).

**Figure 2:**
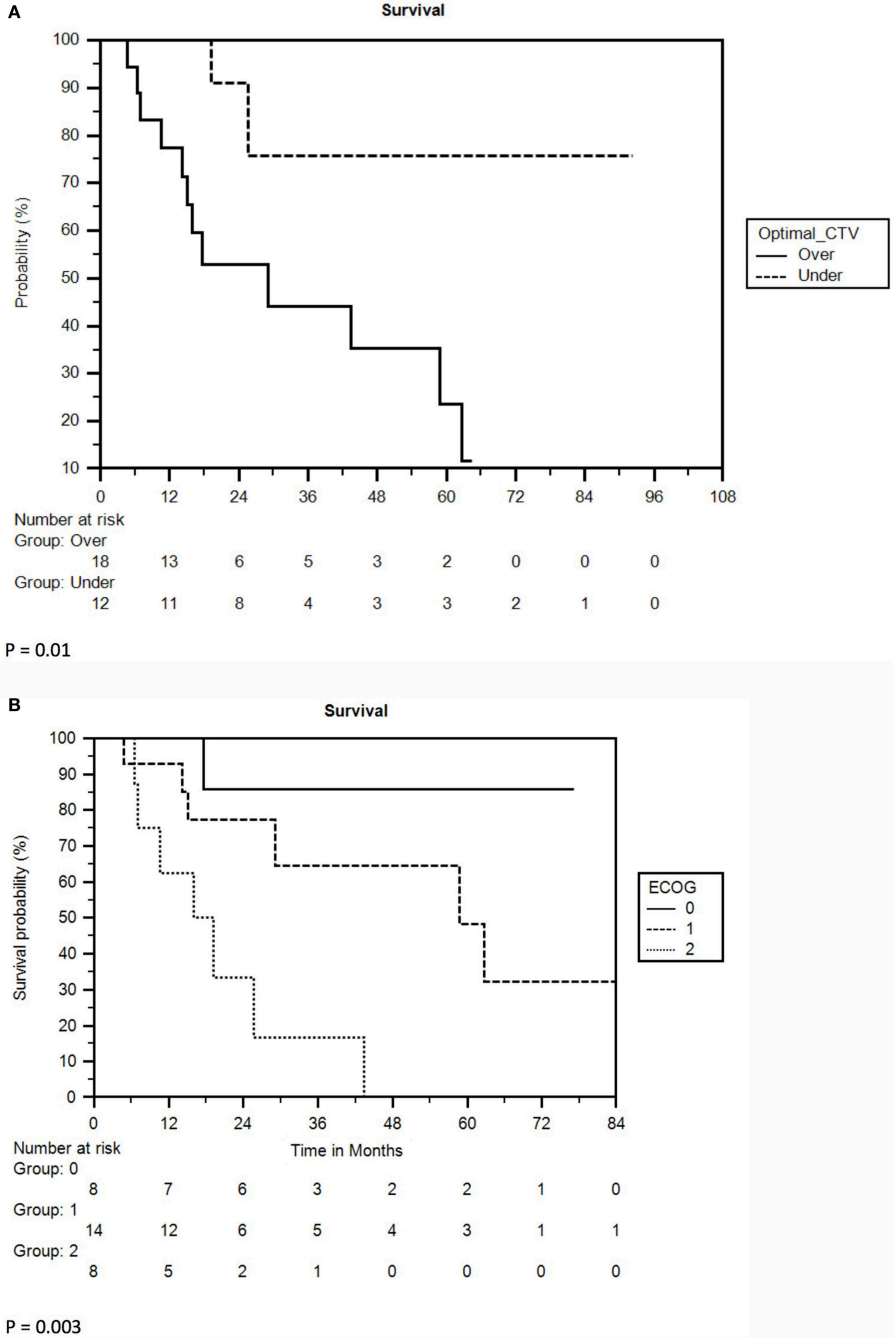
**(A)** Kaplan–Meier survival curve separated by clinical target volume (CTV). Optimal CTV = 24 cc. **(B)** Kaplan–Meier survival curve separated by ECOG performance status.

Multivariate analysis revealed ECOG performance status (PS) and CTV to be independent prognosticators for survival. ECOG PS of 0 yielded a 2-year survival rate of 86%, compared to 77% for ECOG 1 and 33% for ECOG 2 (*p* = 0.01). CTV less than 24 cc yielded 2-year survival of 86 vs. 53% for greater than 24 cc (*p* = 0.005). A full list of prognostic factors with associated KM curves for statistically significant variables in multivariable analysis is reflected in Table 2 and Figure 2.

**Table 2:** **Univariate analysis: survival hazard ratios (*N* = 30)**.

Prognostic factors	*N*	Survival HR (95% CI)	*p*-Value
Serous/clear cell/carcinosarcoma	7	1.68 (0.5–1.6)	0.4
Cervical/endometrioid	23		
SBRT alone^a^	22	2.60 (0.58–11.5)	0.16
SBRT boost	9		
Re-irradiation^b^	13	2.67 (0.88–8.13)	0.08
No re-irradiation	18		
ECOG 2	8	**11.0 (1.63–71.4)**	**0.01**
ECOG 0	8		
ECOG 2	8	**4.20 (1.25–14.1)**	**0.02**
ECOG 1	14		
Central pelvis	10	1.55 (0.46–5.27)	0.48
PA/PSW LN	20		
CTV ≥ 24 cc	18	**4.69 (1.14–19.3)**	**0.03**
CTV < 24 cc	12		
Time to recurrence ≥ 28 mo	15	1.37 (0.47–3.96)	0.56
Time to recurrence < 28 mo	15		
Age ≥ 70	15	0.62 (0.21–1.78)	0.37
Age < 70	15		
Age ≥ 79	10	**3.33 (1.16–9.57)**	**0.03**
Age < 79	20		
BED < 53.6 Gy	15	**3.21 (1.07–9.61)**	**0.04**
BED ≥ 53.6 Gy	15		
Chemo for relapse	13	*2.88 (0.96–8.64)*	*0.06*
No chemo for relapse	16		
Prior chemo	9	*1.98 (0.61–6.43)*	*0.16*
No prior chemo	21		

*CP, central pelvis; PA, para-aortic; PSW, pelvic sidewall; SBRT, stereotactic body radiation therapy; N, number of patients; HR, hazard ratio; CI, confidence interval*.

*Bold indicates statistical significance; italics indicate a trend toward statistical significance*.

*^a^One patient had both a boost and CK alone treated for two separate recurrences*.

*^b^One patient had one site that received external and SBRT boost and another site treated within a previously treated external beam field*.

### Local Control

**Figure 3:**
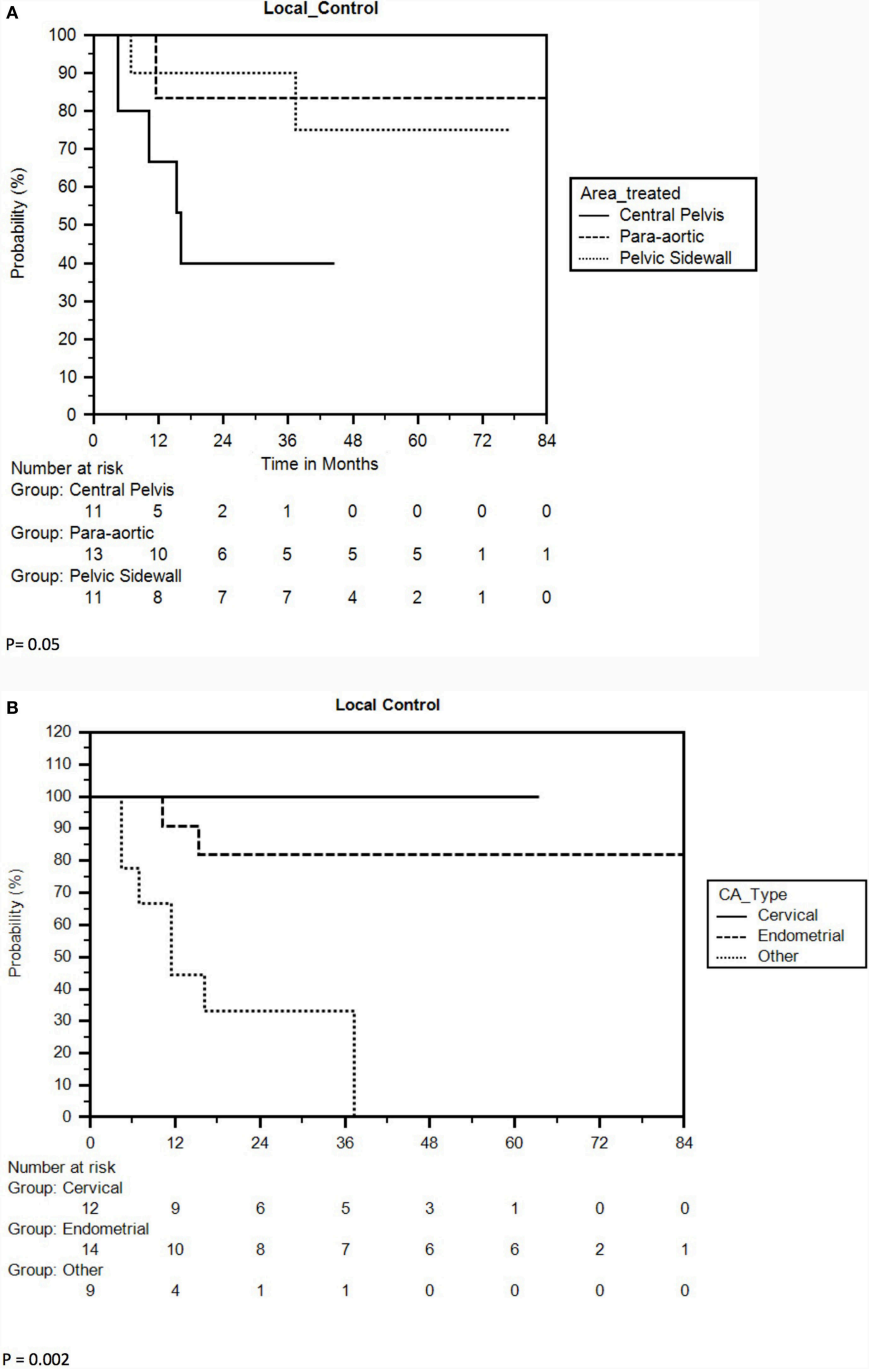
**(A)** Kaplan–Meier local control curve separated by tumor location. **(B)** Kaplan–Meier curve for local control separated by type of primary.

Nine of 35 lesions (26%) failed locally, resulting in LC rates of 80 and 73% at 1 and 2 years, respectively. The 3-year and 5-year LC rates were 73 and 67%, respectively, and median LC could not be calculated due to an inadequate number of recurrences (Figure 1B). Ovarian/non-endometrioid uterine cancers compared to endometrioid uterine/cervical primaries and CP compared to PSW/PAN recurrences were associated with poorer LC. The 2-year LC rate of cervical, endometrioid uterine, and ovarian/non-endometrioid uterine recurrences was 100, 82, and 33, respectively. Five of the 11 cervical cancers were re-irradiated. At 3 years, 40% of CP compared with 83% of PAN and 90% of PSW recurrences were locally controlled (Figures 3A,B). CP location (HR = 4.78, *P* = 0.02) and ovarian/non-endometrioid uterine cancers (HR = 14.12, *P* = 0.002) were each poor prognosticators for LC per multivariate analysis, with type of primary having a greater influence. A complete list of hazard ratios for potential correlates of LC is portrayed in Table 3.

**Table 3:** **Univariate analysis: local control hazard ratios (*N* = 35)**.

Prognostic factors	*N*	Local control HR (95% CI)	*p-*Value
Serous/clear cell/carcinosarcoma	9	**14.12 (2.86–69.54)**	**0.002**
Cervical/endometrioid	26		
SBRT alone	26	3.57 (0.045–28.6)	0.24
SBRT boost	9		
Re-irradiation	15	0.56 (0.12–2.60)	0.46
No re-irradiation	20		
ECOG 2	9	1.08 (0.11–10.49)	0.95
ECOG 0	9		
ECOG 2	9	1.14 (0.14–9.18)	0.90
ECOG 1	17		
Central pelvis	11	**4.78 (1.26–18.18)**	**0.02**
PA/PSW LN	24		
CTV ≥ 30 cc	18	2.73 (0.57–13.05)	0.21
CTV < 30 cc	17		
Time to recurrence ≥ 28 mo	15	2.35 (0.59–9.39)	0.23
Time to recurrence < 28 mo	15		
Age ≥ 70	18	1.43 (0.38–5.30)	0.43
Age < 70	17		
BED < 53.6 Gy	18	1.13 (0.31–4.21)	0.85
BED ≥ 53.6 Gy	17		
Chemo for relapse	13	2.63 (0.54–12.5)	0.23
No chemo for relapse	21		
Prior chemotherapy	9	1.19 (0.34–4.11)	0.78
No prior chemotherapy	21		

*CP, central pelvis; PA, para-aortic; PSW, pelvic sidewall; SBRT, stereotactic body radiation therapy; N, Number of lesions; HR, hazard ratio; CI, confidence interval*.

*Bold indicates statistical significance*.

In addition to seven patients with local failures, seven others developed distant failures following SBRT (47%). The resulting median DFS was 47 months with 1-, 2-, and 3-year DFS of 78, 61, and 52%, respectively (Figure 1C). Distant failure sites include lung (*n* = 2), liver (*n* = 2), bone (*n* = 1), PAN (*n* = 1), and PSW (*n* = 1). Five of the seven distant failures were cervical cancers, one was endometrial, and one was ovarian. No factors were significant for DFS with *p* ≤ 0.05 in multivariate analysis.

### Toxicity

Grade 2 radiation proctitis occurred in one patient 25 months after completing SBRT. Argon beam coagulation therapy was required to treat the rectal bleeding and the patient has been asymptomatic at last follow-up, over 4 years since presentation of rectal bleeding. She received 45 Gy EBRT to the pelvis followed by 14.4 Gy EBRT to the right PSW followed by an SBRT boost to the PSW target of 15 Gy in three fractions. Her rectum received max dose of 54 Gy with external beam radiation and a max dose of 5.2 Gy with SBRT. One patient with 6 cm supravaginal recurrence of carcinoma *in situ* of the cervix (both squamous and adenocarcinoma histologies) treated initially with hysterectomy endured grade 2 cystitis with hematuria 2 years after SBRT, which resolved without intervention. She received 45 Gy EBRT to the pelvis and a subsequent SBRT boost of 25 Gy in five fractions to the GTV located in a supravaginal/cuff position posterior to the bladder measuring 29.8 cc. The volume of bladder treated by EBRT was 375 cc to a mean dose of 45.5 Gy and max dose of 48.3 Gy, followed by a mean and max dose of 4.4 and 26.3 Gy with SBRT. She is 21 months from additional episodes of hematuria.

Another cervical cancer patient who was initially treated with surgery and adjuvant chemoradiation received SBRT and gemzar/cisplatin for a central recurrence and subsequently developed grade 3 rectovaginal and vesicovaginal fistulas 3 months after treatment requiring urinary and bowel divertions. Exam under anesthesia initially demonstrated no pelvic mass but biopsies were initially not done. Subsequent CT scan demonstrated pelvic mass and biopsy proved recurrent squamous cell carcinoma of the rectum and vaginal cuff. She had diffuse metastatic disease to liver and retroperitoneum on CT at that time. As part of the initial treatment, the rectum and bladder received maximum doses of 49 and 48 Gy, respectively. Three years later, SBRT delivered a maximum dose of 28.9 Gy to the bladder and 27.5 Gy to the rectum in five fractions. Since the toxicity occurred initially with no definite evidence of recurrent disease, we have reported this as radiation toxicity. However, we feel that this toxicity is most likely secondary to undiagnosed recurrent disease, which was ultimately discovered, and not SBRT.

## Discussion

This study focuses on gynecologic cancer recurrence treated by SBRT in the most common sites of local/regional recurrence – namely the PSW, PA lymph node regions, and CP/vagina. By contrast, most comparable data sets include primary and recurrent gynecologic malignancies together or included gynecologic recurrence as a subset of multiple recurrent cancers treated by SBRT. Two Korean studies investigated the efficacy of SBRT for uterine cervical recurrences, one experience reflected the outcomes of PA lesions and the other reported on PSW lesions. Choi et al. reviewed 30 patients with PA recurrences treated with 33–45 Gy in three fractions and described a 4-year LC rate of 67.4% and median progression-free survival of 32 months (32). In the PSW study, the 2-year LC and OS rates were 65 and 43%, respectively (33). Kunos et al. published a phase II study of SBRT for gynecological recurrences, of which 33 patients had pelvic lesions. The treatment was 24 Gy in three fractions with a response rate of 68% and median OS of 20.2 months. The overall survival and LC results of this study are very similar to those reported by Choi, Seo, and Kunos, although some prognosticators differed.

Among the factors that influenced survival, PS and target volume were the strongest correlates, both of which were statistically significant with multivariate analysis. Older patients, lower BEDs, lack of salvage chemotherapy, or previously irradiated patients were more likely to have a worse survival in univariate analysis. Similar studies also found re-irradiation and larger targets to be poor prognosticators for survival, although the optimal cutoff size varies. A data set comprised of PSW recurrences delineated a cutoff size of GTV = 30 cc, whereas another study including just PA LN recurrences identified a PTV = 17 cc (32, 33). Our study found no distinction between CP, PAN, and PSW recurrence and found the optimal CTV to be less than or equal to 24 cc. Longer time to recurrence was commonly noted as a positive prognosticator for survival as well, although it was not a significant factor in our data. Pelvic recurrences treated with surgery, chemotherapy, or conventional radiation reported a better prognosis for central lesions compared to PSW or PA regions, although survival rates among those studies were poor overall (34–39). The current series is the first SBRT experience to report the effect of recurrence location on survival in gynecologic malignancies with no difference in survival between CP, PAN, or PSW recurrences. Likewise, type of primary did not affect survival, including the comparatively more aggressive ovarian and papillary serous uterine cancers compared to cervical cancers and endometrioid adenocarcinoma.

The 5-year LC rate of the entire patient population was 67% and therefore median LC duration could not be obtained. Papillary serous (ovarian and uterine) and carcinosarcomatous tumors were more likely to recur locally compared to cervical and endometrioid types. Of the 12 cervical cancers included in the study, there were no local failures after SBRT. CP recurrences were also more likely to have a local recurrence following SBRT compared to PAN or PSW tumors. Other SBRT data reflects nearly identical LC rates for gynecologic recurrences, regardless of the location within the pelvis (32, 33). The lone predictor of local recurrence among those studies was tumor size which was significant for survival but not local recurrence in our database.

The toxicity profile among our patients is consistent with the literature, where every analogous study reports at least one grade 3 or higher toxicity. Seven incidences of enterovaginal fistulas were described among four series (20, 24, 30, 33). Three of these were in the PSW recurrence study by Seo et al., who determined that a *D*_5cc_ < 30 Gy, V40 < 50 cc, or a GTV < 50 cc drastically decreased the risk of developing a fistula (33). The lone fistula in our review was the result of 25 Gy in five fractions delivered to an 88.8 cc central recurrence following previous radical hysterectomy and post-operative 45 Gy IMRT for the initial disease. It is questionable whether our lone grade 3 toxicity should be coded due to radiation since central disease recurrence was ultimately documented. All three significant late toxicities in our series were treated with SBRT for local recurrence adjacent to the rectum or bladder.

## Conclusion

Although limited by a small patient population and the intrinsic selection bias of a retrospective review, our data corroborates the promising survival and control rates of pelvic and PA gynecologic recurrences treated by SBRT. The toxicity profile is low especially with PSW and PAN recurrence; however, caution should be used with bigger centrally recurring tumors as they are in close proximity to the rectum/bladder, increasing the risk for fistulas in particular if the target region received previous radiation. Retrospective series such as this one suggest that SBRT is emerging as the salvage therapy of choice for select recurrent gynecologic malignancies in the pelvis and PA region when brachytherapy is not possible. Prospective trials would help determine ideal patient selection to maximize efficacy and limit toxicity.

## Author Contributions

SH, AR, and RL contributed to writing the manuscript. AH contributed statistical support. KJ, AR, and SH contributed to data entry. SH, AR, RL, KJ, AH, JY, JF, MG, JL, LB, and JN contributed to editing the manuscript.

## Conflict of Interest Statement

JY, JL, RL, and LB have a small percent ownership in Philadelphia Cyberknife. The remaining authors declare no conflict of interest.
